# Long-term outcomes of one single-design varus valgus constrained versus one single-design rotating hinge in revision knee arthroplasty after over 10-year follow-up

**DOI:** 10.1186/s13018-022-03026-3

**Published:** 2022-03-04

**Authors:** Pablo Sanz-Ruiz, Víctor Estuardo León-Román, José Antonio Matas-Diez, Manuel Villanueva-Martínez, Javier Vaquero

**Affiliations:** 1 Department of Traumatology and Orthopaedic Surgery, General University Hospital Gregorio Marañón, Calle Doctor Esquerdo nº 46, 28007 Madrid, Spain; 2 Faculty of Medicine, Complutense University of Madrid, Pza. Ramón y Cajal, Square, University City, 28040 Madrid, Spain; 3 Department of Traumatology and Orthopaedic Surgery, Villalba Hospital, Carretera de Alpedrete a Moralzarzal M-608 Km 41, 28400 Collado Villalba, Spain; 4 Avanfi Institute, Orense 32, 28020 Madrid, Spain

**Keywords:** Revision knee, Instability, Rotating hinge, Condylar constrained knee

## Abstract

**Background:**

The appropriate degree of constraint in knee prosthetic revision is unknown, necessitating the use of the lowest possible constraint. This study aimed to compare the long-term clinical and survival results of revision with rotation hinge knee (RHK) VS constrained condylar constrained knee (CCK) implants.

**Methods:**

Overall, 117 revision case were prospectively reviewed and dividing into two groups based on the degree of constraint used, using only one prosthetic model in each group (61 CCK vs 56 RHK). All implants were evaluated for a minimum of 10 years. Survival of both implants at the end of follow-up, free from revision for any cause, aseptic loosening, and septic cause was compared.

**Results:**

Better results were seen with use of the RHK in joint ranges of (*p* = 0.023), KSCS (*p* = 0.015), KSFS (*p* = 0.043), and KOOS (*p* = 0.031). About 22.2% of the cases required repeat surgery (11.7% RHK vs 29.6% CCK, *p* = 0.023). Constrained condylar implants had a significantly lower survival rates than rotating hinge implants (*p* = 0.005), due to a higher aseptic loosening rate (*p* = 0.031).

**Conclusion:**

Using a specific RHK design with less rotational constraint has better clinical and survival outcomes than implants with greater rotational constraint, such as one specific CCK.

## Background

The increasing number of patients undergoing primary total knee arthroplasty (TKA) has been accompanied by a similar increase in the number of revision knee arthroplasty procedures [1], despite advances in instrumentation [2] and the more widespread use of computer assisted surgery [3], which have optimised length of stay and rehabilitation following primary TKA [4]. Aseptic loosening, infection, and instability are the three most common reasons for revision arthroplasty [5].

A minimally constrained conventional prosthesis can be used during revision knee arthroplasty in most situations [6–8]. However, the presence of severe bone loss and instability may necessitate the use of a more constrained prosthesis, such as condylar constrained knee (CCK) or a rotating hinged prosthesis (RHK). Adequate implant selection is indispensable to restore the function of the knee joint; however, inadequate constraint will lead to instability failure (third most common cause of revision), but excessive constraint may increase the risk of aseptic loosening (first cause of revision) [9]. This has popularized the idea of using the least constraint necessary, which is not always easy to determine [1].

The definition of constraint is determined by the degree of freedom an implant allows [10]. The greater constraint of the RHK over the CCK implant is classically accepted [1] due to the lower number of movements allowed by them. However, due to the existence of an internal/external rotation movement associated with each flexion–extension movement of the knee joint [11–13] and because most CCK implants almost completely restrict this rotational movement (due to the high congruence and trapezoidal shape of the post that limits this rotational movement), more surgeons tend to use hinged implants instead of constrained condylar implants, as they question the lesser constraint of these constrained condylar implants [14–16].

Our objectives are: (1) To compare the survival of two highly constrained implants in a group of patients with long-term follow-up (minimum 10 years of follow-up), (2) to assess and compare the differences, if any, in long-term clinical outcomes between these two types of implants, and (3) to determine if there is a higher rate of aseptic loosening with the use of the more constrained hinged implant. To the best of our knowledge, this is the first article that compares the clinical and functional outcomes of two highly constrained implants, a constrained condylar and a rotating hinge implant (CCK and RHK), with different degrees of constraint in over a 10 years of follow-up period.

## Methods

All patients gave their informed consent before being included in this study. This was retroactive study that reviewed a prospectively collected database and was performed in accordance with the principles of the 1964 Declaration of Helsinki as revised in 2013 and was approved by the research ethics committee of our centre.

Between January 2004 and December 2009, 245 consecutive revision total knee arthroplasties were performed in 231 patients by 4 senior training orthopaedic surgeons in a single institution. Patients undergoing surgery after tumour surgery, periprosthetic fracture, or with prior alterations of the extensor apparatus were excluded. Two groups were defined based on the type of implant used. In Group 1, a constrained condylar implant was used (NexGen LCCK, Zimmer Biomet, Warsaw, IN, USA), while in Group 2, an intracondylar rotating hinge implant (Endomodel, Waldemar Link, Hamburg, Germany) was used. All surgeons had previous experience and implanted both implants. Patients that received an implant other than the two implants in this study (even if it was a CCK or rotating hinge implant) or patients using a LCCK implant with another insert other than the CCK (posterior stabilized) were also excluded.

**Fig. 1:**
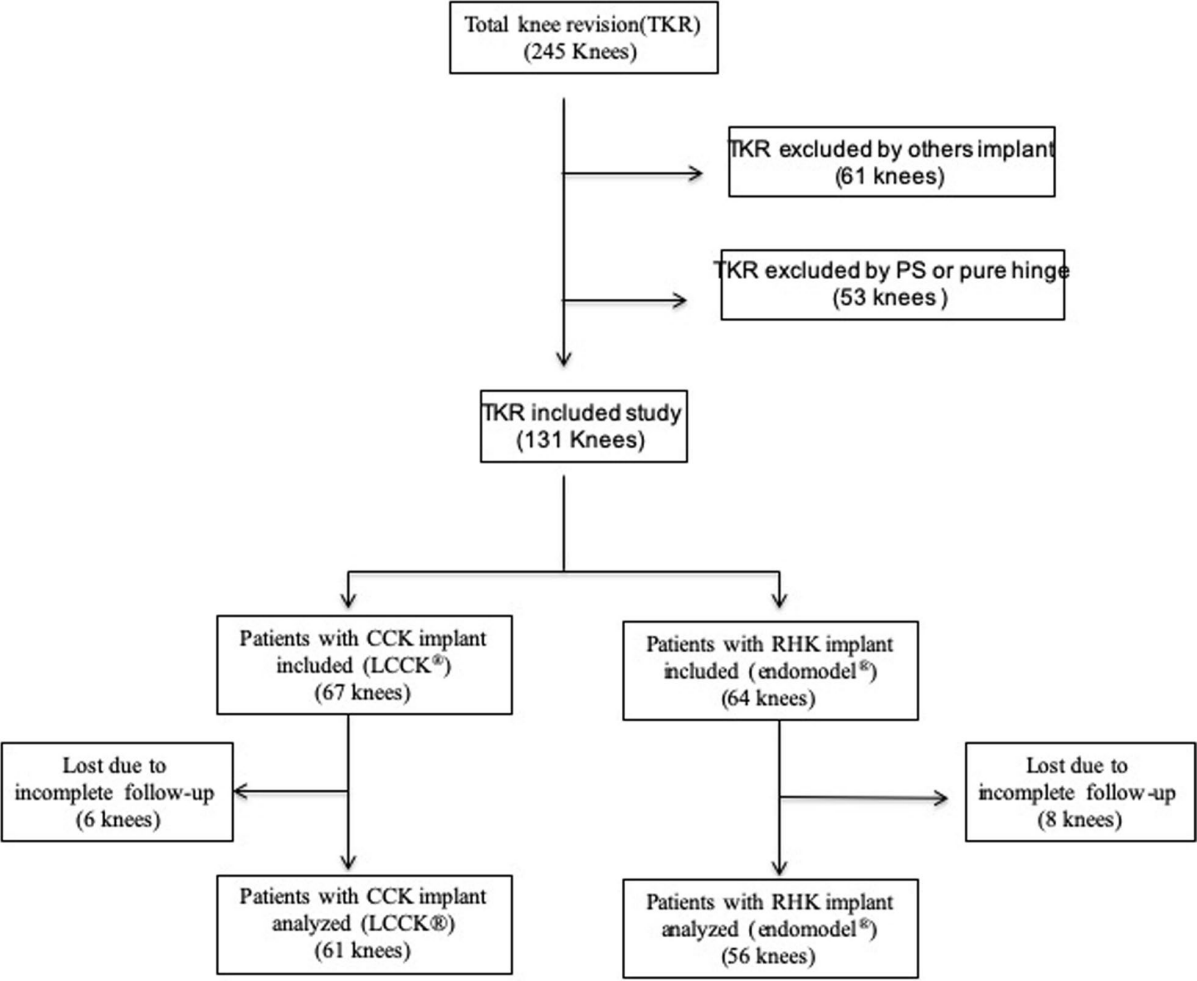
Flow chart of the patients included in the study. TKR: Total knee arthroplasty; PS: posterostabilized; CCK: constrained condylar knee; RHK: rotating hinge knee

A total of 131 knees were included, of which 117 completed a minimum follow-up of 10 years. The indication for the type of implant was at the discretion of the primary surgeon based on the bone defect, ligament status, and their own experience. In cases with absence of collateral ligament, difficulties to equalize gaps or posterior capsule dysfunction a RHK implant was selected. A total of 61 knees were operated with the CCK implant and 56 with the rotating hinge implant (Fig. 1). If severe ligament instability was observed, a rotating hinge implant was selected.

Demographic data were collected for each patient, including age at surgery, sex, reason for revision, prior implant, number of comorbidities expressed as Charlson index, and degree of bone defect according to the Anderson Orthopaedic Research Institute (AORI) Classification[17]. Standard follow-up of each patient includes clinical and radiographic reviews at 3 months, 6 months, one year, and every 2 years thereafter. Any complication or reoperation was recorded at any time during the follow-up. Clinical evaluation was performed using the Knee Society Clinical Score (KSCS), the Knee Society Functional Score (KSFC), and range of motion (ROM). Radiographic evaluation was performed by an orthopaedic surgeon unrelated to the surgery, including under load anteroposterior views, lateral views, and Merchant skyline views in all patients. The presence or location of radiolucence lines was determined according to the modification of the Knee Society TKA radiographic evaluation system for long-stemmed revision prostheses [18]. All data were retrospectively collected from our database.

All procedures were carried out following standard revision TKA procedures. In both cases, conventional stem lengths were used (120 mm in the Endomodel group and 145 mm in the LCCK group). In both groups, fixation of both components was cemented, differentiating pandiaphyseal cementation in patients with rotary hinges, compared to those with metaphyseal cementation using press-fit stems in the group of patients with CCK implants. In AORI III defect trabecular metal metaphyseal cones were used when primary stability was not obtained.

### Statistical analysis

Statistical analysis was performed using SPSS version 24.0 for MacOs (IBM Corp., Armonk, New York, USA). All data were checked for normality using the Shapiro–Wilk W test. Patient demographics were described using medians and standard deviation. The t test was used to compare scores for the normal distribution between the preoperative and postoperative data. Chi-square test and Fisher exact test were used to compare qualitative variables. Statistical significance was set at *p* < 0.05. Prosthesis survival was analysed using the Kaplan–Meier method. Survival analysis was performed using revision due to any reason, aseptic loosening, or septic revision as endpoints, and 95% confidence intervals were calculated.

## Results

The mean follow-up duration was 13 ± 1.475 years. No significant differences were seen in age (*p* = 0.6), sex (*p* = 0.82), body mass index (*p* = 0.54), preoperative comorbidities measured with the Charlson index (*p* = 0.75), and ASA (*p* = 0.81) between both groups. However, patients in the hinged group had poorer preoperative range of motion (*p* = 0.001), greater preoperative varus deformity (*p* = 0.001), lower preoperative KSS (*p* = 0.035), and lower preoperative KOOS (*p* = 0.031). The time between the primary implant and revision was 64.8 ± 38.5 months, and this was lower among patients in the hinged group (61.3 ± 36.9 months vs 67.1 ± 43.6 months, *p* = 0.021), though in this group the percentage of patients with previous revision implants was significantly higher (25% vs 8.1%, *p* = 0.037). The main cause of revision was aseptic loosening (53%), followed by periprosthetic infection (30.7%), with a greater number of septic revisions in the hinged group (39.3%) than in the CCK implant group (22.9%), though this difference was not significant (*p* = 0.11). The demographic characteristics of the patients are summarized in Table 1.

**Table 1:** Patient’s demographic

	General	CCK group	RHK group	*p* value
Age (yr)	75.1 ± 7.3	73.1 ± 6.4	77.4 ± 7.7	*p* = 0.6
Gender (Male/female)	45/74	23/38	20/36	*p* = 0.82
Body mass index (kg/m^2^)	29.4 ± 7.2	28.9 ± 4.3	30.3 ± 6.1	*p* = 0.54
ROM	85.9º ± 19.3	86.7º ± 17.2	84.2º ± 24.9	*p* = 0.001
HKA	8.4º ± 7.7	7.9 ± 5.5	8.9 ± 9.5	*p* = 0.001
Previous revision implant (%)	16.2%	8.1%	25%	*p* = 0.037
Time until revision (month)	64.8 ± 38.5	67.1 ± 43.6	61.3 ± 36.9	*p* = 0.021
Charlson index	2 (0–9)	2 (0–8)	2 (0–9)	*p* = 0.75
*ASA class (%)*				*p* = 0.81
I	0	0	0	
II	48	41	36	
III	62	59	64	
IV	0	0	0	
*Bone defect*				*p* = 0.047
AORI I	8	5	3	
AORI II	75	45	30	
AORI III	34	11	23	
KSCS	38 ± 17.1	39.4 ± 16.6	36 ± 15.1	*p* = 0.035
KSFS	31.8 ± 12.1	32.1 ± 11.5	30.3 ± 13.2	*p* = 0.057
KOOS	40.9 ± 13.1	43.2 ± 15.1	37.4 ± 9.3	*p* = 0.031
*Reason for revision*				*p* = 0.11
Aseptic loosening	53%	60.6%	39.3%	
Infection	30.7%	22.9%	39.3%	
Instability	16.3%	11,5%	21.4%	

ROM, range of motion; HKA, hip-knee angle; ASA, American Society of Anesthesiologists score; KSCS, Knee society clinical score; KSFS, Knee society functional score; KOOS, Knee injury and Osteoarthritis Outcome Score

The mean surgery time was 106.4 ± 27.5 min, and this was significantly lower in patients implanted with a rotating hinge (96 min vs 115 min, *p* = 0.02) (Table 2). No difference was observed in the number of cones used in both group (8 patients; 7 tibial and 6 femoral in CCK group, vs 6 patients; 6 tibial and 3 femoral in RHK group. *p* = 0.24). At the end of the follow-up period, 37.6% of patients experienced some type of complication. These are summarized in Table 3. Aseptic loosening (12.8%), periprosthetic infection (5.1%) and periprosthetic fractures (5.1%) were the most common complications—the number of complications was significantly higher in the CCK implant group (*p* = 0.001) as shown in Table 3.

**Table 2:** Comparison of the results of both groups at final follow-up

	General	CCK group	RHK group	*p* value
ROM	94.3º ± 20.9	93.5 º ± 15	99.1º ± 25.9	*p* = 0.023
HKA	3.4º ± 3.1	3.6 ± 2.4	3.2 ± 3.7	*p* = 0.32
Surgical time (min)	106.4 ± 27.5	115 ± 33.1	96 ± 24.6	*p* = 0.02
KSCS	80.1 ± 10.3	78.7 ± 12.1	83.7 ± 9.8	*p* = 0.015
KSFS	57.4 ± 8.8	56 ± 8.9	58.7 ± 9	*p* = 0.043
KOOS	67.9 ± 15.7	64.1 ± 16	68.5 ± 13.2	*p* = 0.031
Radiolucency’s	29.9%	45.9%	12.5%	*p* = 0.001
New surgery (any cause)	22.2%	29.6%	11.75%	*p* = 0.023
Implant revision	21.1%	27.6%	9.8%	*p* = 0.035

ROM, range of motion; HKA, hip-knee angle; KSCS, Knee society clinical score; KSFS, Knee society functional score; KOOS, Knee injury and Osteoarthritis Outcome Score

**Table 3:** Comparison of complication between patients with constrained condylar knee prostheses and those with rotating hinge knee prostheses

Complication	Implant		
	CCK group	RHK group	Total
Aseptic loosening	12 (19.6%)	3 (5.3%)	15 (12.8%)
PJI	3 (4.9%)	3 (5.4%)	6 (5.1%)
Instability	3 (4.9%)	0	3 (2.6%)
Extensor mechanism instability	2 (3.2%)	3 (5.4%)	5 (4.2%)
Stiffness	0	1 (1.8%)	1 (0.85%)
Synovitis	0	1 (1.8%)	1 (0.85%)
Periprosthetic fracture	5 (8.2%)	1 (1.8%)	6 (5.1%)
Patela tendon lesion	5 (8.2%)	0	5 (4.2%)
Haematoma	2 (3.2%)	0	2 (1.71%)
TOTAL	32/61 (50.8%)	12/56 (21.4%)	44/117 (37.6%)

PJI, periprosthetic joint infection

A significant difference was seen between preoperative and postoperative values in the range of motion, KSS, KSFS, and KOOS. The mean range of motion gain was 9.2° (*p* = 0.001). In patients in whom a hinged implant was used, the joint range of motion achieved (99.1 ± 25.9° vs 93.5° ± 15, *p* = 0.023) as well as the gain in range of motion (14.9° vs 6.8°, *p* = 0.001) was significantly higher than in patients with constrained condylar implants (Table 2). The mean gain in the different clinical scores was 42 (KSS), 25.6 (KSFS), and 27 points (KOOS) (*p* = 0.001). The postoperative KSS results (83.7 ± 9.8 vs 78.7 ± 12.1; *p* = 0.015), KSFS (58.7 ± 9 vs 56 ± 8.9; *p* = 0.043) and KOOS (68.5 ± 13.2 vs 64.1 ± 16; *p* = 0.031) were significantly higher in patients with hinged implants (Table 2).

According to the Knee Society criteria, radiolucencies were observed in 29.9% of patients. Out of these, 23.9% occurred at the tibial level and 17.9% at the femoral level. At the end of follow-up, 15 patients (12.8%) had to undergo repeat surgery for implant loosening. The group of patients with CCK implant had significantly more radiolucencies (45.9% vs 12.5%, *p* < 0.001). (Table 4). At the end of follow-up, a greater number of implants had to be revised in this group for aseptic loosening (*p* = 0.021). Table 4.

**Table 4:** Radiolucency around the implants at final follow-up

	Total	CCK group	RHK group	*p* value
Aseptic loosening	15/117 (12.8%)	12/61 (19.6%)	3/56 (5.3%)	*p* = 0.021
Radiolucency	35/117 (29.9%)	28/61 (45.9%)	7/56 (12.5%)	*p* < 0.001
Femoral radiolucency	21/117 (17.9%)	20/61 (32.8%)	1/56 (1.8%)	*p* < 0.001
AP Tibia radiolucency	28/117 (23.9%)	21/61 (34.4%)	7/56 (12.5%)	*p* = 0.003
Lat tibia radiolucency	23/117 (19.7%)	19/61 (31.1%)	4/56 (7.1%)	*p* = 0.001

**Fig. 2:**
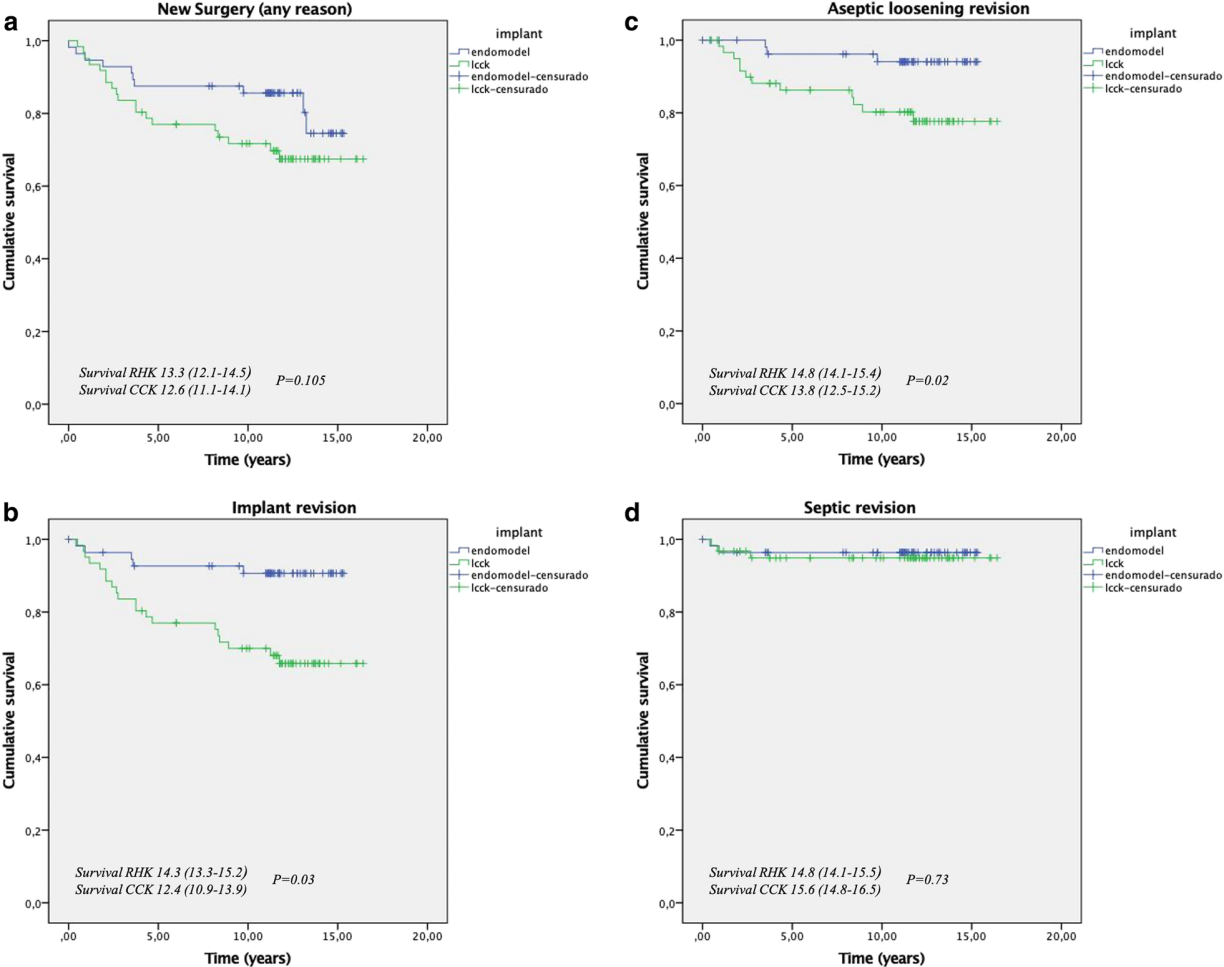
**A** Long-term Kaplan–Meier survival curves comparing CCK implant group and the RHK implant group using any new surgery as the endpoint. **B** Long-term Kaplan–Meier survival curves using implant revision as endpoint **C** Long-term Kaplan–Meier survival curves using aseptic loosening surgery as the endpoint. **D** Long-term Kaplan–Meier survival curves using septic revision surgery as the endpoint

The mean implant survival in both groups at the end of follow-up was 75.2%. No significant differences were seen in survival in both groups when this was defined as the performance of any type of intervention. However, implant survival, defined as the need for repeat surgery to replace the implant, was significantly greater in patients with hinged implants (91.1%) as compared to patients with CCK implants (67.2%) (*p* = 0.003) (Fig. 2A, B). When aseptic loosening was considered as the cause of implant failure, survival was significantly higher in the group of patients with hinged implants (94.6%) than in the group of patients treated with CCK implants (80,3%) (*p* = 0.02) (Fig. 2C). No differences were seen in survival due to sepsis between both groups (96.4% RHK vs 95.1% CCK, *p* = 0.73) (Fig. 2D).

## Discussion

Despite continuous improvements in implants and surgical techniques, the number and complexity of knee revisions continues to increase. The constraint/loosening ratio has relegated the use of theoretically more constrained implants (rotating hinges) in elderly patients with low functional demands, or in situations where another type of implant cannot be used [1, 5, 19]. However, in recent years, based on the good results obtained with specific models of rotating hinges in primary and revision surgery [20–24], some authors have questioned this marginal use of rotating hinges [25, 26]. This allows physiological rotation with flexion/extension, restricted by most CCK implants. Unfortunately, all attempts to compare these two types of implants were unable to show differences in either of these characteristics, probably because of the high number of different implants available (particularly in the hinge group), the short follow-up period, and the existence of a high heterogeneity in the different groups. To the best of our knowledge, this is the longest follow-up series ever reported that compared a single constrained condylar implant and a single hinged implant.

In this series, we observed a poorer preoperative score on the different scales, as well as a lower range of motion in the group of patients that underwent surgery with hinged implants, despite the lack of differences in comorbidities before surgery. The higher percentage of cases of septic origin and severe instability may also explain the presence of these poorer preoperative outcomes [27]. Hossain et al. [28] compared the mid-term results of 343 knee revisions performed with 3 different types of constraint (PS, CC and RH). In their population, patients undergoing surgery with RH implants had a lower range of motion (1°) and KSCS score (3 points); their findings are similar to those seen in this series (2.6° of ROM, 3 points on KSCS and 2 points on KSFS).

One of the main limitations to the widespread use of hinged implants is the theoretical increased risk of complications observed with their use. Pour et al. [5] reported medical complications rates of 18% and surgical complications rate of 20.9% after prosthetic revision in 43 knees with a third-generation condylar hinged implant, recommending its use only in selected cases. However, the indications selected were 23 massive bone defects, 10 instabilities, 4 periprosthetic fractures, and three comminuted supracondylar fractures, these cases had high complexity which could involve bias. To the best of our knowledge, there is no study that directly compares the complication rate between hinged and semi-constrained implants in a homogeneous group of patients. Shen et al. [1] found no increase in the rate of infections or aseptic loosening with the use of hinges in AORI II and III defects in septic or aseptic revisions. Similar data were recently been published by Malcon et al. and Yoon et al. [27, 29]. Our results differ from those of these authors, who found a significantly higher complication rate with the use of semi-constrained implants (CCK) (32/61) as compared to rotary hinged implants (12/56). After excluding minor complications (haematoma or partial lesions of the extensor apparatus), the complication rate remained higher in patients in whom semi-constrained implants were used as a result of a greater number of aseptic loosening (19.6% vs 5.3%), ligament instability (4.9% vs 0%), and periprosthetic fractures (8.2% vs 1.8%) (Table 3).

There is considerable controversy regarding the difference in clinical outcomes between hinge implants and semi-constrained CCK implants. Walker et al. [30] in one of the first articles published on this subject, questioned the theoretical clinical superiority of CCK implants over RHK implants. The clinical results of 56 RHK and 33 CCK implants were subsequently compared, and lower residual laxity was seen with RHK implants, and this led to better results in the KSCS, but had no impact on the range of motion or KSFS result. Hossain et al. [28] in 2010 found a greater range of motion with the use of hinged implants (111.7° vs 106°) but Dwivedi et al. [31] did not observed any difference in ROM, OKS, and KSS. However, their mean follow-up duration was less than 5 years. Malcon et al. [27] in the first published meta-analysis analysing 544 CCK vs 254 RHK implants, and recently Yoon Jung-Ro et al. [29] with 775 CCK vs 402 RHK implants, reported that no differences were found in the postoperative range of motion between both types of implants; however, both authors observed a minimal higher postoperative clinical score in the CCK group, but in this group, the preoperative clinical score was also higher. In our series, a significantly greater range of motion and better clinical results (KSCS, KSFS, and KOOS) were seen with the use of RHK implants at the end of follow-up. This observed difference in the range of motion can be explained by the intrinsic characteristics of the RHK implant chosen (intracondylar hinge, in which collateral ligaments are sectioned for the correct biomechanics of the design, etc.) which makes it easier to achieve a high degree of mobility [20, 26]. The clinical differences observed between this series and previous studies may be explained by the high homogeneity of both groups, using only one implant per group versus the high heterogeneity in other series that mixed different implants with completely different biomechanical designs.

Along with the theoretical increased risk of complications, the fear of decreased implant survival due to its theoretical greater constraint is the second limiting factor for expanded use of rotating hinges. Gehrke et al. [20] and Samguietei et al. [26] showed a 90% and 93.3% survival in complex primary and revision surgery, respectively, with the same RHK implant after 13.5 years of follow-up. To the best of our knowledge, all studies published to date have been unable to find survival differences between semi-constrained and constrained implants [1, 14, 22, 27–29, 31–35]. In our series, we observed a lower survival of the semi-constrained implant versus the constrained implant (67.2% vs 91.1%), mainly due to the greater number of revisions for loosening and instability. The high level of radiolucencies at the end of follow-up in CCK implants (45.9%) is particularly striking compared to those in patients with hinged implants (12.5%), which in our opinion could reflect the deleterious effect on implant fixation of the high degree of rotational constraint of certain CCK implants (Table 4).

This study has some limitations. First, patients were operated on by different surgeons. However, they were all experienced surgeons, with the same philosophy as regards selection of the degree of constraint. Second, the low number of patients in each group may have influenced the lack of statistical significance in some of the results observed. However, the high homogeneity of both groups, as only a single prosthetic implant was used, allowed us to observe statistically significant differences despite this low number of patients. Third, the results observed in this study can only be extrapolated to the included implants, due to their special mechanical characteristics (high rotational constraint in LCCK and intracondylar rotating hinge design in Endomodel). However, the choice of these implants was based on the existence of good results with each of them [20, 36] and their wide use in different countries, being the most commonly used implants in their group according to different registries [37, 38]. Fourth, we used two different fixation technique, full cemented stem in RHK group and hybrid cemented (metaphyseal cemented + press-fit diaphyseal stem), and this may explain the differences observed in aseptic loosening rate. However, there are a lot of reports [39–41] that show similar results using both techniques with the same bone defect and similar implant. In our series, no difference was observed in the number of cones used in both groups to treat severe bone defect. Fifth, the patients included in the group of constrained implants had poorer clinical outcomes and a greater number of surgeries and infections, which could affect the results obtained. However, despite this “limitation” or possible bias, patients with hinged implants had better results and survival at the end of follow-up.

## Conclusion

Our results suggest that the use of one specific, more constrained implant, such as an intracondylar rotating hinge, does not worsen clinical outcomes, but has better results and longer long-term survival. We hypothesize that this difference could be the result of the lower rotational constraint of these implants; however, more experimental and clinical test are necessary to support this affirmation.

## Data Availability

The datasets used and analyzed during the current study are available from the corresponding author on reasonable request.

## Abbreviations

RHK: Rotation hinge knee
CCK: Condylar constrained knee
KSCS: Knee Society Clinical Score
KSFS: Knee Society Functional Score
KOOS: Knee Injury and Osteoarthritis Outcome Score
TKA: Total knee arthroplasty
ROM: Range of motion
AORI: Anderson Orthopedic Research Institute
KSS: Knee Society Score
PS: Postero stabilized
CC: Condilar constrained
RH: Rotating hinge
ASA: American Society of Anesthesiologists Score
HKA: Hip-knee angle
